# Black Sea Mussels Qualitative and Quantitative Chemical Analysis: Nutritional Benefits and Possible Risks through Consumption

**DOI:** 10.3390/nu14050964

**Published:** 2022-02-24

**Authors:** Magdalena Mititelu, Sorinel Marius Neacșu, Eliza Oprea, Denisa-Elena Dumitrescu, Mirela Nedelescu, Doina Drăgănescu, Teodor Octavian Nicolescu, Adrian Cosmin Roșca, Manuela Ghica

**Affiliations:** 1 Department of Clinical Laboratory and Food Safety, Faculty of Pharmacy, University of Medicine and Pharmacy Carol Davila, 020956 Bucharest, Romania; magdalena.mititelu@umfcd.ro; 2 Professional Farma Line, 116 Republicii Street, 105200 Baicoi, Romania; sorinel.neacsu@pfarma.ro; 3 Department of Organic Chemistry, Biochemistry and Catalysis, Faculty of Chemistry, University of Bucharest, 030018 Bucharest, Romania; 4 Microbiology Department, Faculty of Biology, University of Bucharest, 1-3 Portocalilor Way, 060101 Bucharest, Romania; 5 Department of Organic Chemistry, Faculty of Pharmacy, “Ovidius” University of Constanta, 6, Căpitan Aviator Al Șerbănescu Street, 900470 Constanta, Romania; 6 Department of Hygiene and Environmental Health, Faculty of Medicine, Carol Davila University of Medicine and Pharmacy, 8 Eroii Sanitari Blvd, 020956 Bucharest, Romania; mirela.nedelescu@umfcd.ro; 7 Department of Food Hygiene and Nutrition, National Institute of Public Health, National Centre for Environmental Hazards Monitoring, 1-3 Dr. Leonte Street, 020956 Bucharest, Romania; 8 Department of Pharmaceutical Physics and Informatics, Faculty of Pharmacy, Carol Davila University of Medicine and Pharmacy, 6, Traian Vuia Street, 020956 Bucharest, Romania; doina.draganescu@umfcd.ro; 9 Department of Organic Chemistry, Faculty of Pharmacy, University of Medicine and Pharmacy Carol Davila, 020956 Bucharest, Romania; teodor.nicolescu@umfcd.ro; 10 Department of Drug Analysis, Biopharmacy and Biological Medicines, Faculty of Pharmacy, “Ovidius” University of Constanta, 6, Căpitan Aviator Al Șerbănescu Street, 900470 Constanta, Romania; 11 Department of Biostatistics, Faculty of Pharmacy, University of Medicine and Pharmacy Carol Davila, 020956 Bucharest, Romania; manuela.ghica@umfcd.ro

**Keywords:** mussels, biochemical compounds, nutritive quality, total lipid extract, polyunsaturated fatty acids, heavy metals, estimated daily intake, bioconcentration factor

## Abstract

Mussels have a particular nutritional value, representing a highly valued food source and thus sought after worldwide. Their meat is a real culinary delicacy, rich in proteins, lipids, carbohydrates, trace elements, enzymes, and vitamins. The seasonal variation of mussels’ biochemical composition has been studied to determine the best harvesting period to capitalize on various biologically active fractions. In this work biochemical determinations have been performed on fresh flesh samples of *Mytilus galloprovincialis* specimens from the Black Sea coast to study seasonal variations in mussels’ biochemical compounds. An analysis of significant lipid classes and the fatty acid composition of lipid extracts obtained from mussel flesh has also been performed. Since mussels retain pollutants from the marine environment, in parallel, the concentration of heavy metals in the meat of mussels collected for the analysis of the chemical composition was investigated. The impact and risk of heavy metal poisoning due to food consumption of mussels contaminated due to pollution of the marine harvesting area was evaluated by the bio-concentration factor of metals and estimated daily intakes of heavy metals through mussel consumption.

## 1. Introduction

*Mytilus galloprovincialis* is one of the most widespread invertebrates in the Black Sea, whose rich content in valuable nutrients has brought it to researchers’ attention. The remarkable nutritional quality of mussel flesh is due to the proportion of vital components necessary for human nutrition: protein, fat, carbohydrates, macro- and micronutrients, enzymes, vitamins, etc. Mussels are considered authentic delicacies, along with shrimp, lobster, crab, cuttlefish, octopus, and other seafood [1,2]. The first to use the medicinal properties of mussels were the Chinese. Even today, mollusk meat is considered a medicine used especially in oncological, cardiovascular, ocular, and gynecological diseases [3,4,5,6].

Mussel meat is known for its nutritional value due to the high quality of proteins, the presence of essential free amino acids, and water-soluble and fat-soluble vitamins. The total content of free amino acids reaches an average of 0.7% in fresh meat, and of these, 40% is represented by the fraction of essential amino acids [7,8]. Fresh mussel meat contains the same high-quality protein as red meat but has much less total fat, saturated fat, and almost 25% fewer calories. Thus, replacing red meat with mussel meat provides a low-calorie diet that benefits weight management. Like fish, seafood is highly sought after for the dietary nature of meat (low energy value) and particularly high content of minerals, especially zinc, selenium, etc. [9,10,11]. The abundance of essential amino acids, vitamins, and minerals makes mussel meat a valuable functional food for strengthening the immune system. As a result, nutritionists recommend the inclusion of seafood in general in diets to enhance immunity, especially in the current epidemiological context coupled with the increase in global pollution and even many drugs that affect the immune system [12,13,14].

Mussels have superiority in the marine environment in terms of biosynthesis of hydro and fat-soluble vitamins; remarkable is the presence of vitamins D group, which can reach up to 1% of the dry matter, and vitamin B_12_, which comes 6–9.7 μg/g dry matter, much more than in pork containing 1.3 μg/g [4,9]. However, variability in the biochemical composition of mussels may result in variability of nutritional value and flavor. The mussel is an important dietetic source of polyunsaturated ω-3 fatty acids, as eicosapentaenoic acid and docosahexaenoic acid: both are beneficial for health (for example, in the treatment and prevention of cardiac ischemia) [15,16]. Farmed marine mussels from the *Mytilidae* family, especially genera *Mytilus*, are essential for the human diet in providing high levels of proteins, omega-3 polyunsaturated fatty acids, fat-soluble vitamins, and carbohydrates. In recent years, the functional properties of mussel lipids have been investigated, and few dietary supplements based on lipid extracts of mussels have been presented at the market [17,18]. Essential fatty acids have beneficial effects on the heart and blood vessels. These good fats lower blood cholesterol levels, have anti-inflammatory and mild antihypertensive effects, help improve heart function and play an essential role in brain development [19,20,21].

Mussels are found in a wide variety of habitats from areas affected by the tide to completely submerged areas with a range-wide range of temperatures and salinity. They feed on phytoplankton and organic matter by constantly filtering the water sea and are therefore consistently grown in rich areas in plankton. Water quality is a significant factor in the growth of mussels. Mussels can retain and concentrate in their body pollutants from the marine environment and food elements. The most dangerous pollutants that get into the meat of mussels are heavy metals, pesticides, and pathogenic bacteria [22,23,24,25].

Mussels’ biochemical composition is influenced by geographical area, depth, nourishment, gender, age, reproductive status, climate factors, and environmental pollution level. Therefore, mussels harvested from contaminated water sources have an increased risk of infection and chemical poisoning. In addition, consumption of mussels contaminated with heavy metals, such as mercury, cadmium, or lead, can increase the risk of neurological damage and congenital disabilities [26,27,28,29]. Aquatic organisms exposed to marine contaminants such as heavy metals accumulate these elements. Sometimes, the amount of metals relative to the body mass of these organisms increases with the evolution of the food chain. In the case of fishing activities in the contaminated aquatic area and, subsequently, by consuming the catches, the toxic elements are transferred to the human body, where they can cause different organ-specific syndromes (hematological, neurological disturbances, with attention-deficit even at low concentrations of pollutants, cardiovascular diseases, renal and gastrointestinal impairments [30,31,32,33]. The severity of symptoms depends on the metal accumulated and the amount collected. The rate of bioaccumulation is influenced by certain factors such as temperature and condition physiological function of the body (sex, age, size). Heavy metals can create random bonds with cellular biomolecules such as enzymes or proteins to form complexes that may compromise their structure and function when ingested in excessive amounts.

In the present study, a series of determinations were made on mussels harvested from the Romanian Black Sea coast: analysis of seasonal variation of biochemical composition in order to identify the optimal harvesting time for nutrient recovery and analysis of heavy metals concentration in order to determine the safety of consumption.

## 2. Materials and Methods

### 2.1. Analysis of Seasonal Biochemical Composition Variation

**Figure 1:**
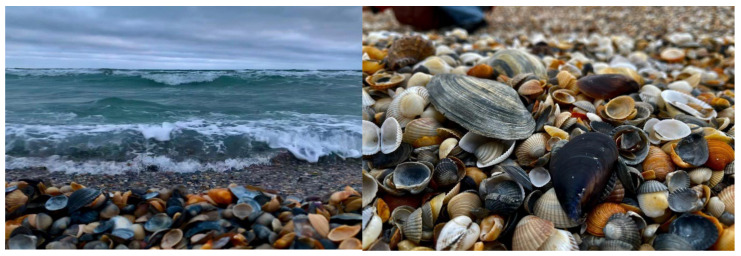
*Mytilus galloprovincialis* from Romanian seaside.

The biochemical determinations have been performed on fresh flesh samples of *Mytilus galloprovincialis* specimens collected during 2020 in the harbor area (A1) near the Port of Tomis Constanta during all seasons: winter (January), spring (April), summer (July) and autumn (October) of the Romanian seaside (Figure 1) in order to evaluate the seasonal variations of the nutrients in the meat composition. In addition, for comparison purposes, analysis has also been performed on specimens collected from the harbor area (A1), the beach area (A2) near Costinesti Beach and the industrial area (A3) near Navodari of the Romanian seaside in autumn in order to evaluate the compositional differences determined by the habitat. Incidentally, the weight distribution on the components—flesh, juice, shells—also been examined.

Proteins have been measured by the Kjeldahl method [32], whereas the Christie method [33] has been used for fats and the orcinol-sulphuric acid method for assaying total carbohydrates [34,35]. Mineral residue has been measured by means of dry tissue calcination at 550 °C. The overall biochemical composition has been expressed in %, in relation to dry weight.

### 2.2. Analysis of the Total Lipid Extract Isolated and Purified from Mussel

For isolation and purification of total lipid extract, adult specimens of *Mytilus galloprovincialis* were used, collected from the Romanian Black Sea coast. First, the flesh was separated from the shells following rinsing under running water. Then, the fat fraction was isolated and purified from the mussel meat by a simple technique [35].

The main classes of lipids were separated and purified by column chromatography on silica gel (60G). After specific treatment, the fatty acids in the lipid fractions were then analyzed by gas chromatography with a model 17 GC gas chromatograph (Shimadzu, Kyoto, Japan) provided with a flame ionization detector and a capillary column.

The process for separation of the significant lipid fractions consisted of the following steps:

(1) Freshly diced and homogenized tissue (500 g) was treated with 800 mL chloro-form-methanol (2: 1 *v*/*v*) mixture using the Soxhlet method for total lipids extraction;
(2) Following extraction of the lipid fraction, the extraction solvent was removed by concentration in a 4001 rotary evaporator (LABOROTA, Schwabach, Germany);
(3) The total lipid extract was further purified by treatment with a mixture of chloroform: methanol: 0.9% KCl solution (10:10:9 *v*/*v*); The lower layer was next retained and concentrated by rotary evaporation, thus obtaining the purified lipid extract;
(4) The purified total lipid extract was treated with 100 mL of acetone and incubated for 24 h at 4 °C, then filtered, retaining both the precipitate and the filtrate;
(5) The precipitate was dissolved in chloroform, using 0.002% butylated-hydroxytoluene (BHT, Sigma-Aldrich, St. Louis, MO, USA) as an antioxidant: this fraction (fraction “a”) contains polar lipids;
(6) The filtrate was evaporated in vacuum and redissolved in n-hexane, with 0.002% BHT as an antioxidant, thus yielding fraction “b”, containing neutral lipids;
(7) The polar lipids in fraction “a” were separated by column chromatography on silica gel (60G Merck, Darmstadt, Germany), and residual neutral lipids were removed by elution with chloroform, yielding fraction no. 1 (containing glycolipids) by elution with acetone and fraction no. 2 (containing phospholipids) by elution with methanol;
(8) Column chromatography on silica gel (60G) was used to separate neutral lipids in fraction “b”. At the same time, hydrocarbons and pigments were removed by elution with n-hexane, and the fatty acid methyl esters were removed by elution with a mixture of n-hexane: diethyl ether (70:5 *v*/*v*). Fraction no. 3 was thus obtained (containing triglycerides) by elution with chloroform, as mobile phase.
(9) According to the Romanian Pharmacopoeia, the tenth edition (R Ph X) [36], the total lipid fraction isolated and purified from the mussel meat was characterized by analyzing acidic, esterification, saponification, and iodine indexes.

Analysis of fatty acids in the total lipid extract isolated and purified from the mussel flesh proceeded as follows: an internal standard (C23:0 methyl ester; Nuchek Prep Inc., Elysian, MN, USA) was added to a lipid extract sample; next, the mixture was dried under nitrogen atmosphere and then subjected to hydrolysis using a 7.9% KOH solution in methanol. After cooling, the samples were treated with a 20% boron trifluoride solution in methanol. Fatty acid methyl esters were subsequently analyzed with the Shimadzu model 17 GC gas chromatograph. The carrier gas used was helium. Assays were injected at 125 °C, following which temperature was set to increase to 170 °C at a 5 °C/min rate and maintained at this level for 4 min. Next, the temperature was again set to rise to 175 °C, at a 0.5 °C/min rate, and finally to 220 °C, at a 4 °C/min rate, when maintained for 3 min. Injector and detector temperature was maintained at 260 °C. The peak area was processed using the Shimadzu Class GC-10 software.

All samples were tested three times, and the results were expressed as means ± SD (standard deviation). A standard mix of fatty acids methyl esters (Nuchek Prep Inc., Elysian, MN, USA) was used to calibrate gas chromatography and determine response factors. All reagents used (Sigma-Aldrich) were of analytical purity.

### 2.3. Analysis of the Heavy Metals from Mussels

Analysis of the heavy metals has been performed on fresh flesh samples of *Mytilus galloprovincialis* specimens collected in all seasons during 2020 (spring, summer, autumn, and winter) from the harbor area (A1), the beach area (A2) and the industrial area (A3) of the Romanian seaside.

To analyze heavy metals (cadmium, copper, chromium, nickel, lead, zinc), mussel, water, and sediment samples were harvested from three areas of the Romanian coastline (A1, A2, and A3) to determine the optimum area for harvesting of least polluted raw material. Water samples, including surface water and bottom water, were collected from 0.5 m below the surface and 2.0 m above the bottom, and sediment samples were about 5 to 10 cm thickness of the surface marine sediment. In total, 12 samples of mussels, water, and sediment were collected from each analyzed area.

Heavy metals analysis was done using the digestion method followed by atomic absorption spectroscopy [37,38]. First, mussels collected were thoroughly rinsed, separated from the shells, grated and dried at 80 °C until constant weight, and then mortar-powdered and homogenized. Next, both powdered samples (shells and flesh, each separately) were mineralized by the wet digestion method: 1 g of each homogenized sample was introduced into the digestion vessel (DK-6 Heating Digester, Velp, Shanghai, China), together with 10 mL 65% HNO_3_, 5 mL 37% HCl and 2 mL 35% H_2_O_2_. Then, the mixture was heated gradually (at 150 °C, for 1 h, 200 °C, for 2 h, 250 °C, for 1 h and 300 °C, for 2 h). Next, the solutions were cooled to room temperature and transferred into a 25 mL volumetric flask, brought to volume with ultra-distilled water.

Aqueous samples (500 mL) were filtered using Whatman No. 41 (0.45 μm pore size) filter paper to estimate dissolved metal content. The filtrate and as-collected water samples (500 mL each) were preserved with 2 mL nitric acid to prevent the precipitation of metals. Both samples were tenfold concentrated on a water bath at 80 °C until the volume reached 50 mL and subjected to nitric acid digestion using the microwave-assisted technique, setting the pressure at 30 bars and power at 700 Watts [39]. After cooling, each sample filtered by filter (Whatman filter, 0.45 μm, Merck). The filtrate was diluted by deionized water to a final volume of 50 mL.

Sediment samples were dried at 80 °C until constant weight and then were mineralized by the wet digestion method, just like the mussel samples.

The resultant solutions were analyzed with a Shimadzu AA 6300 (air/acetylene flame) atomic absorption spectrophotometer in order to determine the heavy metals concentration: cadmium (λ = 228.8 nm), copper (λ = 324.7 nm), zinc (λ = 213.9 nm), chromium (λ = 359.3 nm), lead (λ = 217 nm) and nickel (λ = 232 nm). A blank digestion solution was made for comparison. A standard solution for each element under investigation was prepared and used for calibration. Triplicate determinations were performed for each metal. Results are expressed as triplicate analysis mean ± S.D (standard deviation). Data were statistically evaluated.

All reagents used for the detection of heavy metals in real samples (Merck) were of analytical purity, standard stock metal solutions containing 1000 mg of metal/mL (Titrisol, Merck). All solutions were prepared using distilled deionized water or double deionized.

The linear correlation coefficients (R2), limits of detection (LODs) and limits of quantification (LOQs) for each metal analyzed are presented in Table 1.

### 2.4. Risk Characterisation to Consumer’s Health

To assess the impact and risk of heavy metal poisoning due to food consumption of mussels contaminated due to pollution of the marine harvesting area, the daily intake and the bio-concentration factor of metals were also calculated.

#### 2.4.1. Bioconcentration Factor

Bio-concentration Factor (BCF) can be calculated by the following equation [40,41]:

BCF = C/Cw

where C is the contaminant concentrations in the organisms (μg/kg), Cw is the contaminant concentration in the water (μg/L).

#### 2.4.2. Estimated Daily Intake (EDI)

This parameter is obtained using the following equation [42]:

EDI = C × Con/Bw

where C is the metal levels in mussels (μg/kg), Con is the daily average consumption of mussels (kg person^-1^/day), and Bw represents the average body weight of an adult (kg). We assumed a daily mussel consumption of 3.5 g/person, which is the average quantity consumed in the European Union [43]. Intake estimates were expressed as per unit body weight (mg/kg body wt./weekly and daily).

#### 2.4.3. Hazard Quotient (HQ)

This parameter is calculated for each contaminant using the following equation:

HQ = EDI/RfD

where EDI is the estimated daily intake (mg/kg/day) and RfD (mg/kg/day) is the approximation of daily tolerable exposure to which a person is expected to have any significant risk of harmful effects during a lifespan [44,45].

If the HQ value is less than 1, consumption of mussels does not pose an adverse health hazard to the exposed population in terms of the studied heavy metals. If the HQ is higher than 1, estimated daily intake exceeds the RfD revealing that there is a potential health risk associated with that contaminant.

### 2.5. Statistical Analyses

Statistical analysis was implemented using the open-source software R (R version 4.1.1.) [46]. The basic descriptive statistics like mean, standard deviation and 95% confidence intervals for heavy metals concentrations are reported numerically in its tables. Our input data follow a 4 × 3 balanced design where the factors involved are sample (mussel shell, mussel meat, seawater and sediment) and area (area 1, area 2 and area 3). In verifying our dataset, we perform a Shapiro-Wilk test for normality and Levene’s test to check the homoscedasticity of groups. For every sample, we investigate if there are any statistically significant differences between the means of heavy metal concentration for area levels. Thereby, to make a reliable decision we apply one-way ANOVA parametric test if both conditions are met or Welch’s ANOVA for unequal variances. Otherwise, we perform a Bootstrap version of one-way ANOVA [47]. To high-light which specific group’s means are different we use post hoc Tuckey tests for multiple comparisons. Statistical significance level was considered at alpha 5% (*p* < 0.05).

## 3. Results

### 3.1. Seasonal Biochemical Composition Variation of Mussel

**Figure 2:**
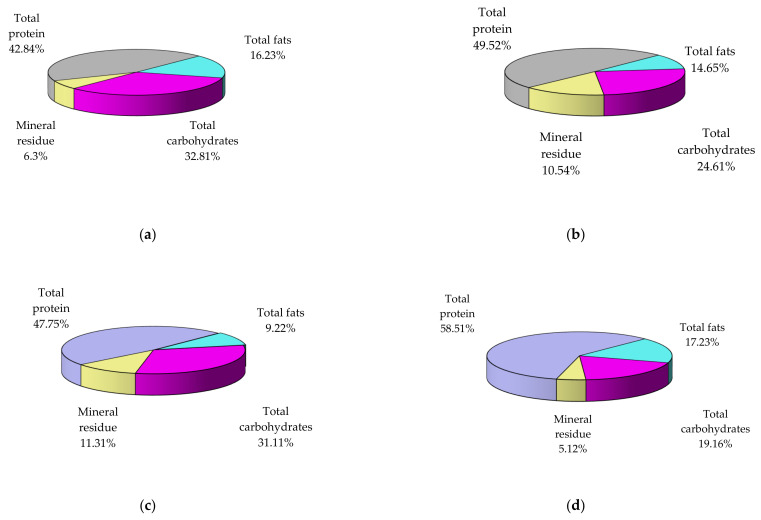
The seasonal variations in biochemical composition of mussel flesh collected from A1 area, the harbor area near Port of Tomis (dry basis, %): autumn (**a**), winter (**b**), summer (**c**), spring (**d**).

A summary of results obtained concerning seasonal variations in mussel flesh composition is shown in Figure 2 and Table 2. In general, data observed showing maximum concentrations (protein, fat) in spring and during pre-breeding, and minimum concentration after breeding, in autumn. Instead, maximum concentration of carbohydrates occurs in autumn.

**Figure 3:**
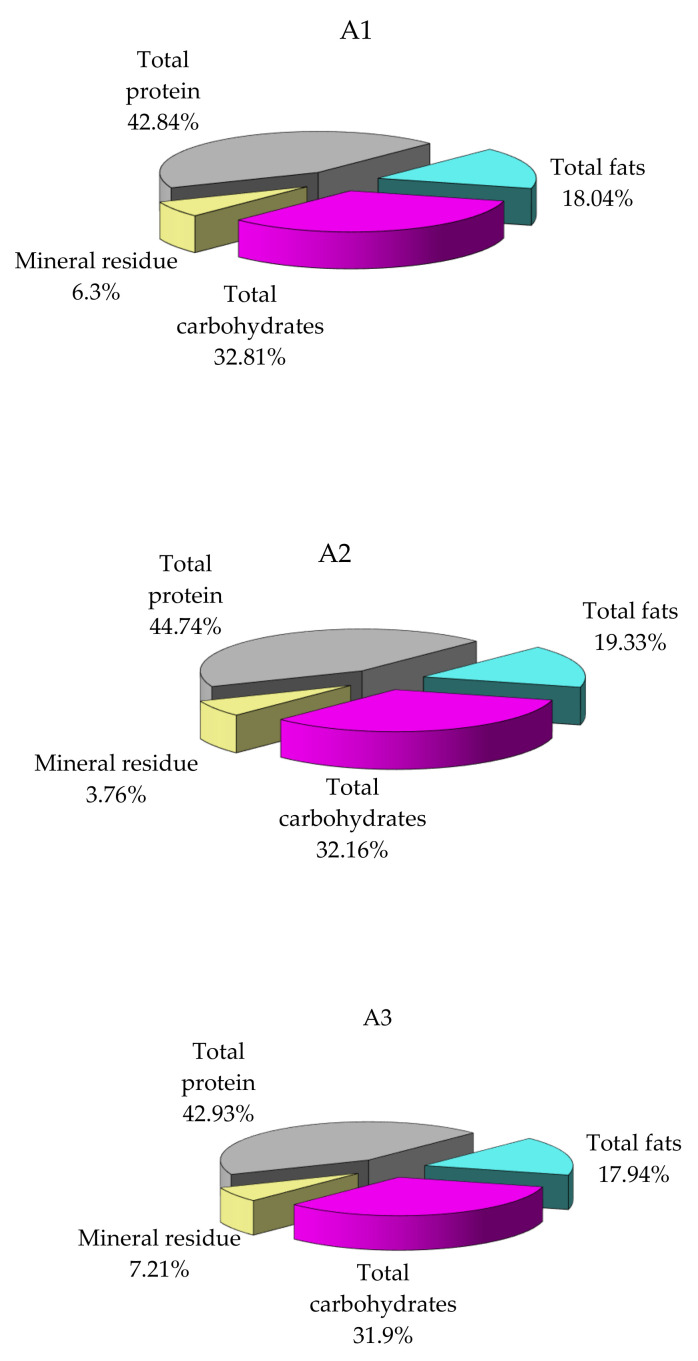
Variation in concentrations of the main classes of compounds in the *M. galloprovincialis* flesh specimens collected from different sites (% dry sample). A1 area, the harbor area near Port of Tomis, the beach area (A2) near Costinesti Beach and the industrial area (A3) near Navodari.

Three lots of mussel specimens of the same age, collected from A1, A2 and A3, respectively, showed significantly different biochemical composition values (Figure 3).

Specimens collected from the area A2 display much higher protein and lipid content and approximately similar carbohydrate content, although the analysis was performed at the same time of the year (Figure 3). Differences may be attributable to specific area food conditions, which advocates for the importance of environmental factors influence. There are no significant differences in the composition of mussel samples collected from areas A1 and A3.

Weight percentage ratios of the main components resulted after cleavage of adult mussel specimens in relation to fresh weight are as follows:

- flesh: 21.14–27.56%;
- juice: 15.76–18.94%;
- shells: 53.5–63.1%.

Mussel flesh is well-known for its nourishing value resulted from the content rich in high quality protein, presence of certain essential free amino-acids as well as of water and lipid soluble vitamins. At the same time, mussel flesh is the core of the main physiological and biochemical changes determined by physiological cycles and the influence of the marine environment. Juice is the part generally lost in processing, whereas shells are the major component part in terms of weight.

### 3.2. Composition of Total Lipid Extract from Mussel

**Figure 4:**
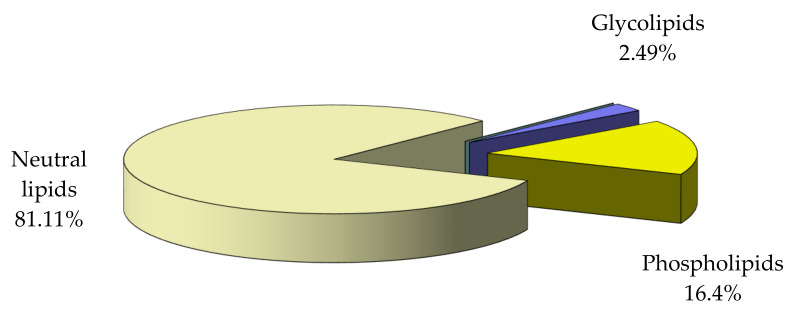
Distribution of main lipid classes relative to total lipid in *M. galloprovincialis* flesh.

Examination of distribution of the main lipid classes in total lipid (Figure 4) shows that the dominant fraction consists of neutral lipids.

- The total lipid extract represents 14.32% of dry tissue.
- The total fatty acids are 69.27% of the total lipid.

**Figure 5:**
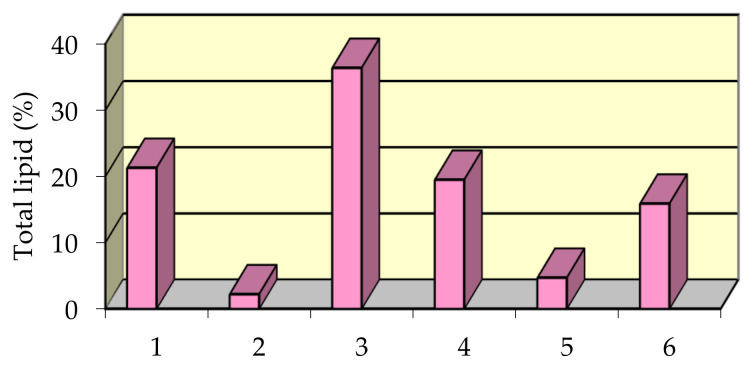
Lipid fractions distribution in mussel total lipid extract: Phospholipids (1); Glycolipids (2); Triglycerides (3); Methyl esters of fatty acids (4); Hydrocarbons and pigments (5); Other lipids (sterols, fatty alcohols, fatty acids etc.) (6).

Percent distribution of separate lipid fractions in the total lipid extract is shown in Figure 5.

Table 3 indicates the values of the examined parameters, characteristic to the total lipid extract isolated and purified from mussel flesh. The high iodine value is notable, indicating high unsaturation of the fatty acids contained in the total lipid extract.

Percent distribution of fatty acids in the total lipid extract isolated from *Mytilus galloprovincialis* is shown in Table 4. Fatty acids were encoded according to number of carbon atoms, number and position of double bonds. Thus, Table 4 highlights the high concentration ω-3 fatty acids as compared to ω-6 fatty acids (ω-3/ω-6 = 6.14). At the same time, of note is the high concentration of polyunsaturated fatty acids in comparison with saturated and unsaturated fatty acids, accounting for the high unsaturation indicated by the iodine value for mussel total fat fraction. Among fatty acids, the most abundant are myristic acid (C14:0), palmitic acid (C16:0), palmitoleic acid (C16:1ω-7), oleic acid (C18:2ω-6) and eicosapentaenoic acid (C20:5ω-3).

### 3.3. Heavy Metals Concentration from Mussels and Seawater

Metal concentrations in mussel meat samples decrease in the order: Zn > Cu > Cr> Ni > Pb > Cd. Mean metal concentrations with their standard deviations and confidence intervals are shown in Table 5. The results showed that the content of heavy metals is higher in mussel samples collected from the industrial area than those from the harbor and beach area. The heavy metal content of meat mussels ranged between: Cd 0.19–0.63 μg/g, Cu 5.27–11.18 μg/g, Zn 8.57–14.45 μg/g, Cr 4.84–7.2 μg/g, Pb 0.68–1.84 μg/g, Ni 1.18–5.34 μg/g. The results were lower than those observed in a previous study and similar with findings from other Black Sea regions [37,42,48].

The amount of lead in the analyzed mussel meat samples exceeded the maximum allowable levels established by the Commission Regulation (EC) no. 1881/2006 [49] in the case of the samples collected from A3 (with industrial activity). Cadmium levels were within the normal range in all mussel samples. No thresholds for copper, zinc, nickel or chromium have yet been set for these marine foods.

**Figure 6:**
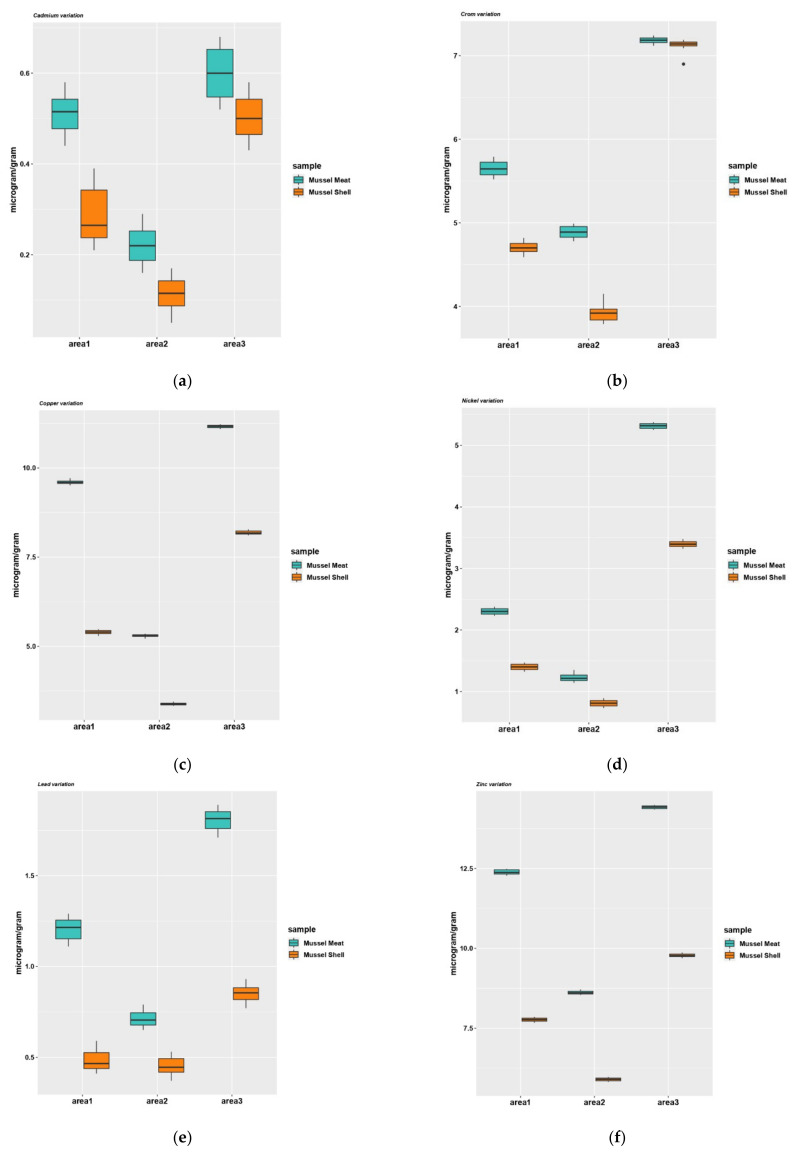
The metal levels detected in mussels (*Mytilus galloprovincialis*) meat and shell from the Black Sea: cadmium (**a**), chromium (**b**), copper (**c**), nickel (**d**), lead (**e**), zinc (**f**). Area1, the harbor area near Port of Tomis, the beach area (area2) near Costinesti Beach and the industrial area (area3) near Navodari.

The results showed the same distribution of heavy metal content in mussel shell samples (Zn > Cu > Cr> Ni > Pb > Cd) as in the mussel meat sample (Figure 6). The mean concentration of heavy metals in mussel shell samples with their standard deviations are given in Table 6. The amount of heavy metals in mussel shells varied within the following limits: Cd between 0.08–0.53 μg/g, Cu between 3.35–8.21 μg/g, Zn between 5.86–9.82 μg/g, Cr between 3.85–7.16 μg/g, Pb between 0.41–0.88 μg/g and Ni between 0.77–3.43 μg/g. No exceedances of the maximum permissible levels of heavy metals were noted.

A post-hoc Tukey test for multiple comparisons was performed at the level of each heavy metal analyzed (cadmium, copper, chromium, nickel, lead, zinc) depending on the type of location (Area 1, Area 2, Area 3) and sample (mussel meat, mussel shells) and it was found that in all cases both main effects are statistically significant (Figure 6). Post-hoc tests were performed for all pairwise comparisons of heavy metal level averages taking into account the complete set of interactions. There was statistical significance among all samples analyzed (*p* < 0.0001), but a lower level of heavy metals was observed in the shell samples compared to meat samples collected from the same area. Experimental data indicate a high concentration of heavy metals in all samples collected from Area 3 compared to the other areas, the lowest values being recorded in the samples from Area 2. In the meat samples collected from Area 3, the values of the lead concentration exceed the maximum allowable level.

The results of seawater and sediment investigation in terms of heavy metal content indicate higher concentrations in sediments than in the seawater samples.

The content of Cd, Cu, Zn, Cr, Pb and Ni in seawater ranged between 8.65–15.26 μg/L, 5.27–39.05 μg/L, 48.15–64.23 μg/L, 56.32–75.46 μg/L, 18.40–29.24 μg/L, 27.21–46.27 μg/L, respectively. Corresponding concentrations in sediment samples ranged between 10.6–22.42 μg/g, 40.98–91.99 μg/g, 86.99–154.07 μg/g, 70.93–94.10 μg/g, 17.91–38.10 μg/g, 28.90–56.13 μg/g.

The highest concentrations of heavy metals were observed in seawater and sediment samples collected from industrial area (A3), followed by harbor area (A1) and beach area (A2) (see Table 7 and Table 8). Zinc and lead exceeded the maximum allowable levels in seawater from harbor and industrial area, while cadmium was exceeded in sediment samples picked-up from industrial area. No exceedances were recorded for the other investigated metals.

### 3.4. Estimated Risk to Consumers

#### 3.4.1. Bioconcentration Factor

**Figure 7:**
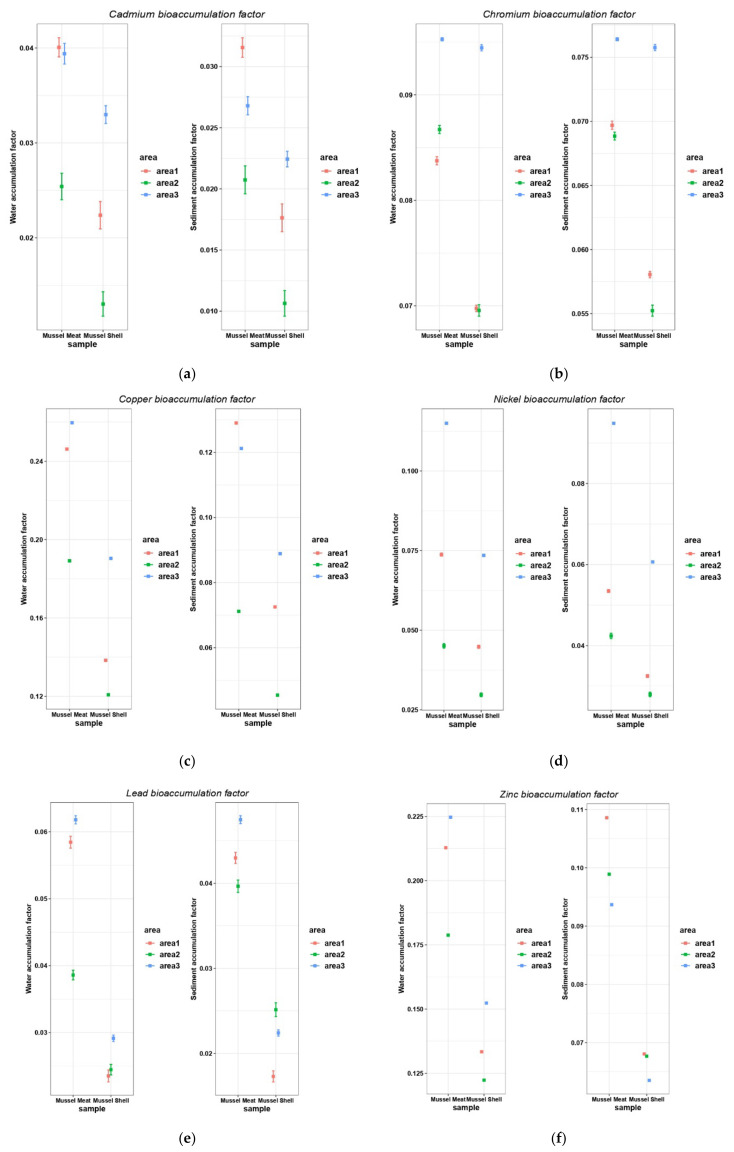
The metal bioconcentration factors related to water and sediment samples in studied areas for cadmium (**a**), chromium (**b**), copper (**c**), nickel (**d**), lead (**e**), zinc (**f**). Area1, the harbor area near Port of Tomis, the beach area (area2) near Costinesti Beach and the industrial area (area3) near Navodari.

The analysis of the heavy metal transfer from water and sediments to mussels shows that bio-concentration factors vary by study area and by anatomical part of mussels (Figure 7).

Copper and zinc presented the highest accumulation levels in mussel meat collected from the harbor (A1) and industrial areas (A3). Cadmium, lead and copper were transferred in a less proportion from the aquatic environment to mussel bodies. In all studied areas, less heavy metals accumulated in mussel shells than mussel meat.

#### 3.4.2. Estimated Risk through Mussel Consumption

Estimation of potential risks by investigating estimated daily intakes of heavy metals through mussel consumption was the subject of several studies, inclusive located on the Black Sea coasts [42,51,52,53].

Calculated EDIs and HQs for each contaminant thorough mussel consumption in study areas are presented in Table 9. The highest values for estimated daily intakes were observed in industrial area, in which we also recorded the highest values of HQ, followed by harbor area and beach area. Even if HQs were below the critical value 1, so no possible risks are associated with mussel consumption collected from the study areas, we noted the case of lead which registered the highest calculated HQs from all heavy metals: 0.2 in A1, 0.03 in A3, 0.012 in A2, respectively.

Our results are in accordance with findings of other studies conducted in order to assess potential human risks associated with mussel consumption in the region of Black Sea [54,55,56]. It should be noted also that calculated hazard quotients for investigated heavy metals within the current research were higher than values found in other investigated food categories in Romania (honey, food supplements) [57,58].

## 4. Discussion

There are people with a long tradition of seafood consumption worldwide (Caribbean, Asia, Mediterranean area, Scandinavian peninsula). Specialists unanimously accept that constant consumption of fish and seafood brings multiple health benefits. In general, people with a long tradition of consuming seafood have had easy access to marine resources [59]. Unfortunately, there are also exceptions, such as the Romanian people, possible due to influences of others peoples, whose food base is supported mainly by proteins from the meat of terrestrial animals [60,61].

Mussels are rich in high biological value proteins lipids rich in essential fatty acids, vitamins, and minerals. Therefore, mussels are among the most popular and cultivated seafood. There are many countries where culture mussels are produced in large quantities and consumed by the local population, and used for export: China, Chile, Japan, the Republic of Korea, the United States of America, Spain, etc. [62].

Data obtained from the analysis of mussel biochemical composition and their main compounds classes (proteins, fats, carbohydrates, minerals) and significantly related to the seasonal composition variation are helpful to determine the best harvesting period for capitalization of the various active fractions biologically. The main categories of nutrients, depending on the season, vary within the following limits in the analyzed samples (% dry sample): total proteins (42.84–58.51), total carbohydrates (12.22–32.81), total fats (9.22–17.23), mineral residue (5.12–11.31).

Experimental data showed an increased protein (58.51%) and lipid (17.23%) content in spring specimens, according to similar studies [27,63]. Therefore, to benefit from the valuable proteins in the composition of mussel meat, it is recommended to consume the samples harvested in spring, when their content is maximum in proteins rich in essential amino acids and lipids, or winter (49.52%).

It is well-known that the proteins from mussels have a remarkable biological value due to their high content of easily digestible essential amino acids, which leads to increased assimilation of essential amino acids. Furthermore, with a high concentration of vitamins and oligo-minerals, the constant consumption of proteins from mussel meat can significantly improve health, especially by strengthening the immune system [1,4].

It is noteworthy that the concentration of carbohydrates and minerals in the spring is minimal compared to the other four seasons, which could be an advantage for people who, for various reasons, should benefit from a hypoglycemic diet and/or poor in electrolytes. In addition, in the analyzed mussel samples, it can also be seen that in autumn, they accumulate a large amount of carbohydrates (32.81%, the highest of the four seasons), but also of fats (16.23%, the second-largest value after spring), while proteins (42.84%) have the lowest values. As a result, in the case of people with significant metabolic imbalances, it might be beneficial to include only mussels harvested at certain times of the year in their diet.

Analysis of fatty acids composition of the main lipid fractions showed high concentrations of polyunsaturated fatty acids in neutral lipids (also representing the dominant fraction, 81.11%), which gives them a particular biological value. The total fatty acids are 69.27% of the total lipid in analyzed mussels.

Analysis of the total lipid extract isolated and purified from mussel flesh showed abundant polyunsaturated fatty acids content, particularly of the ω-3 group fatty acids, as compared with saturated and unsaturated fatty acids, which accounts for the high degree of unsaturation indicated by the iodine value (82.34) for the fat fraction in mussel flesh.

Among fatty acids, the most abundant in the analyzed samples are myristic acid, palmitic acid, palmitoleic acid, oleic acid, and eicosapentaenoic acid. Experimental data indicate a report ω-3/ω-6 of 6.14. The 4.96 ratio of polyunsaturated to saturated fatty acids also shows an increased content of unsaturated lipids in mussel meat.

Even if the lipid fraction in mussel meat is not found in high concentrations, 9.22–17.23% dry sample, the composition rich in polyunsaturated fatty acids (especially in ω-3 group fatty acids) makes it particularly valuable for consumers’ health [64].

The beneficial effect of long-chain ω-3 polyunsaturated fatty acids in the composition of mussel meat has been highlighted by clinical studies that have shown significant improvements in the serum lipid profile of people who regularly included mussel meat in their diet and especially at the patients with cardiovascular disease. There has even been an improvement in consumers’ health with a 20% reduction in the risk of sudden cardiac death [65,66].

Due to their filtering capacity, mussels are recognized as marine organisms with an increased potential for accumulating contaminants in the aquatic environment. Therefore, rigorous investigations are needed regarding the content of various pollutants in mussel meat intended for consumption [67].

Estimated daily intake rates of metals through consumption of mussels harvested from the Romanian Black Sea were below the daily tolerable limits recommended by the Joint FAO/WHO Expert Committee on Food Additives: 0.001 mg/kg bw for cadmium, 0.5 mg/kg bw for copper, 1 mg/kg bw for zinc, 0.14 mg/kg bw for chromium, and 0.0035 mg/kg bw for lead [44,45].

The vast majority of the investigated mussel samples did not exceed allowable values for metals ions recommended by Commission Regulation (EC) no. 1881/2006 [49], and there are no concerns about their consumption, especially when calculated hazard quotients were below the critical value 1 for all analyzed heavy metals, meaning no possible risks are associated with mussel consumption from the study areas. However, Pb concentrations in mussel meat samples taken from area 3 have slightly overrun, which could be attributed to anthropogenic changes/impacts accordingly to Catianis [68]. Over the years, previous studies of the Black Sea mussels have also reported slightly excessive Pb concentrations in that area [37,69], and the evolution trends were not very clear due to large concentration fluctuations [69]. Moreover, the accumulation of the metals in the gills, visceral mass, and remaining tissues of *M. galloprovincialis* was measured [70], while other studies discussed the biomagnification process of copper, lead, nickel, and chromium, which was observed at small fish (trophic level 3) in some areas [71].

There are scientific studies that highlight the possibility of capitalizing on shells, not just mussel meat [72,73]. As a result, the analysis of contaminants in the shells is also critical.

Although pollution is quite low according to our experimental results and does not affect consumers’ health, there are still differences between the areas investigated in terms of the level of pollutants. In areas with intense human activity, permanent monitoring is required of analyzed pollutants and an extension of the tested parameters (mercury, aromatic hydrocarbons, pesticides, microplastics, etc.).

## 5. Conclusions

Mussels are an essential source of valuable nutrients (proteins, fats, carbohydrates), and their composition is influenced by seasonal factors and the quality of the environment in which they grow. Consequently, it is essential to analyze the influence of these factors to optimize the harvesting conditions of these marine species to benefit from specimens rich in nutrients and poor in contaminants.

In spring specimens, experimental data showed an increased protein (58.51%) and lipid (17.23%) content. Therefore, to benefit from the valuable proteins in the composition of mussel meat, it is recommended to harvest in spring when its content is maximum, or winter (49.52%). At the same time, the samples from spring present the advantage of containing a minimum level of carbohydrates and minerals, while in autumn, they accumulate a large amount of carbohydrates (the highest of the four seasons), and fats (the second-largest value after spring), with low protein concentration. As a result, in the case of people with significant metabolic imbalances, it might be beneficial to include only mussels harvested at certain times of the year in their diet.

Analysis of fatty acids composition of the main lipid fractions showed high concentrations of polyunsaturated fatty acids in neutral lipids (also representing the dominant fraction, 81.11%), which gives them a particular biological value. Among fatty acids, the most abundant in the analyzed samples are myristic acid, palmitic acid, palmitoleic acid, oleic acid, and eicosapentaenoic acid, with a 4.96 ratio of polyunsaturated/saturated fatty acids and a report ω-3/ω-6 of 6.14.

The vast majority of the investigated mussel samples did not exceed recommended allowable values for metals ions [49], so there are no concerns about their consumption, especially as the calculated hazard quotients were below the critical value 1 for all analyzed heavy metals.

## Figures and Tables

**Table 1:** Linear correlation coefficients (R^2^), Limits of Detection (LOD) and Limits of Quantification (LOQ) for heavy metals.

Element	R^2^	LOD (µg/g)	LOQ (µg/g)
Cadmium	0.997	1.6 × 10^−3^	18 × 10^−3^
Copper	0.985	54 × 10^−3^	172 × 10^−3^
Zinc	0.993	51 × 10^−3^	167 × 10^−3^
Chromium	0.995	52 × 10^−3^	169 × 10^−3^
Lead	1.000	44 × 10^−3^	152 × 10^−3^
Nickel	0.998	46 × 10^−3^	158 × 10^−3^

**Table 2:** Annual limits of biochemical composition variation in mussel flesh (% dry sample).

Components	Variation Limits (%)
Total proteins 9	42.84–58.51
Total fats	9.22–17.23
Total carbohydrates	12.22–32.81
Mineral residue	5.12–11.31

**Table 3:** Values of examined parameters relative to the *Mytilus galloprovincialis* total lipid extract.

Parameter	Value ± SD
Iodine value (g I_2_/100 g fatty acids)	82.34 ± 0.66
Acid value (mg KOH/g sample)	38.11 ± 0.33
Saponification value (mg KOH/g sample)	180.95 ± 0.25
Ester value (mg KOH/g sample)	147.17 ± 0.50

SD—standard deviation.

**Table 4:** Percent distribution of fatty acids in the total lipid extract isolated from *Mytilus galloprovincialis*.

Fatty Acid	mg/g ± SD (%)
C12:0	0.06 ± 0.13
C14:0	8.23 ± 0.55
C14:1	1.28 ± 0.32
C15:0	0.56 ± 0.11
C16:0	19.15 ± 0.54
C16:1ω-7	7.35 ± 0.74
C17:1	0.43 ± 0.16
C18:0	3.85 ± 0.65
C18:1ω-7	4.35 ± 0.32
C18:1ω-9	5.81 ± 0.84
C18:2ω-6	3.18 ± 0.52
C18:3	2.15 ± 0.21
C18:4ω-3	0.32 ± 0.14
C20:1	1.79 ± 0.48
C20:3	0.11 ± 0.04
C20:4ω-6	0.68 ± 0.18
C20:5ω-3	14.46 ± 0.68
C22:1	0.41 ± 0.14
C22:5ω-3	0.49 ± 0.16
C22:6ω-3	8.44 ± 0.31
Σ saturated fatty acids	31.85
Σ ω-3	23.71
Σ ω-6	3.86
Σ monounsaturated fatty acids	6.17
Σ polyunsaturated fatty acids	30.62
ω-3/ω-6	6.14
Polyunsaturated fatty acids/Saturated fatty acids	4.96

SD—standard deviation.

**Table 5:** Heavy metals concentrations (μg/g) in mussel meat samples.

Elements	Samples N = 12	Mean	SD	Lower	Upper	MAL μg/g [49]
				CI 95%	CI 95%	
Cd	Area1	0.5100	0.0445	0.4775	0.5424	1
	Area2	0.2208	0.0420	0.1952	0.2464	
	Area3	0.5991	0.0574	0.5642	0.6341	
Cu	Area1	9.5966	0.0612	9.5564	9.6368	-
	Area2	5.2980	0.0377	5.2739	5.3220	
	Area3	11.1609	0.0416	11.1333	11.1884	
Zn	Area1	12.3883	0.0734	12.3436	12.4330	-
	Area2	8.6133	0.0528	8.5786	8.6449	
	Area3	14.4225	0.0488	14.3927	14.4522	
Cr	Area1	5.6483	0.0896	5.5938	5.7028	-
	Area2	4.8900	0.0743	4.8447	4.9352	
	Area3	7.1841	0.0375	7.1601	7.2089	
Pb	Area1	1.2050	0.0633	1.1664	1.2435	1.5
	Area2	0.7125	0.0455	0.6824	0.7411	
	Area3	1.8050	0.0618	1.7649	1.8460	
Ni	Area1	2.3033	0.0526	2.2713	2.3353	-
	Area2	1.2291	0.0665	1.1818	1.2680	
	Area3	5.3158	0.0452	5.2883	5.3433	

Note: N—number of collected and analyzed samples from each area; SD—standard deviation; CI—confidence interval; MAL—Maximum allowable level; A1 area, the harbor area near Port of Tomis, the beach area (A2) near Costinesti Beach and the industrial area (A3) near Navodari.

**Table 6:** Heavy metals concentrations (μg/g) in mussel shell samples.

Elements	Samples N = 12	Mean	SD	Lower	Upper	MAL μg/g [49]
				CI 95%	CI 95%	
Cd	Area1	0.2850	0.0634	0.2394	0.3224	1
	Area2	0.1133	0.0386	0.0883	0.1389	
	Area3	0.5016	0.0495	0.4715	0.5317	
Cu	Area1	5.3991	0.0583	5.3624	5.4357	-
	Area2	3.3816	0.0373	3.3521	3.4078	
	Area3	8.1850	0.0541	8.1491	8.2198	
Zn	Area1	7.7641	0.0588	7.7283	7.7999	-
	Area2	5.8933	0.0529	5.8611	5.9255	
	Area3	9.7800	0.0554	9.7395	9.8204	
Cr	Area1	4.7041	0.0698	4.6562	4.7517	-
	Area2	3.9225	0.1063	3.8546	3.9762	
	Area3	7.1233	0.0769	7.1108	7.1668	
Pb	Area1	0.4850	0.0638	0.4347	0.5250	1.5
	Area2	0.4516	0.0504	0.4185	0.4851	
	Area3	0.8516	0.0482	0.8189	0.8850	
Ni	Area1	1.3991	0.0517	1.3676	1.4306	-
	Area2	0.8100	0.0551	0.7764	0.8435	
	Area3	3.3975	0.0527	3.3625	3.4319	

Note: N—number of collected and analyzed samples from each area; SD—standard deviation; CI—confidence interval; MAL—Maximum allowable level; A1 area, the harbor area near Port of Tomis, the beach area (A2) near Costinesti Beach and the industrial area (A3) near Navodari.

**Table 7:** Heavy metals concentrations (μg/L) in sea water samples.

Elements	Samples N = 12	Mean	SD	Lower	Upper	MAL μg/L [50]
				CI 95%	CI 95%	
Cd	Area1	12.7325	0.0482	12.6994	12.7650	20
	Area2	8.6900	0.0525	8.6551	8.7248	
	Area3	15.2150	0.0627	15.1781	15.2599	
Cu	Area1	38.9999	0.0731	38.9466	39.0532	100
	Area2	5.2980	0.0377	5.2739	5.3220	
	Area3	11.1609	0.0416	11.1333	11.1884	
Zn	Area1	58.2166	0.0862	58.1596	58.2712	50
	Area2	48.1908	0.0545	48.1550	48.2230	
	Area3	64.1966	0.0586	64.1609	64.2323	
Cr	Area1	67.4441	0.1299	67.3580	67.5291	100
	Area2	56.3950	0.1112	56.3273	56.4626	
	Area3	75.4175	0.0682	75.3699	75.4580	
Pb	Area1	20.6291	0.0837	20.5782	20.6800	20
	Area2	18.4600	0.0933	18.4032	18.5167	
	Area3	29.2125	0.0534	29.1795	29.2477	
Ni	Area1	31.2375	0.0880	31.1839	31.2910	50
	Area2	27.2600	0.0717	27.2124	27.3075	
	Area3	46.2200	0.0717	46.1622	46.2778	

Note: N—number of collected and analyzed samples from each area; SD—standard deviation; CI—confidence interval; MAL—Maximum allowable level; A1 area, the harbor area near Port of Tomis, the beach area (A2) near Costinesti Beach and the industrial area (A3) near Navodari.

**Table 8:** Heavy metals concentrations (μg/g) in sediment samples.

Elements	Samples N = 12	Mean	SD	Lower	Upper	MAL μg/g [50]
				CI 95%	CI 95%	
Cd	Area1	16.1625	0.0696	16.1171	16.2162	20
	Area2	10.6516	0.0708	10.6046	10.6993	
	Area3	22.3691	0.0882	22.3154	22.4228	
Cu	Area1	74.4090	0.1132	74.3317	74.4862	150
	Area2	41.0691	0.1218	40.9863	41.1578	
	Area3	92.0908	0.1488	91.9956	92.1715	
Zn	Area1	114.0958	0.1636	113.9962	114.1954	150
	Area2	87.0975	0.1716	86.9930	87.2019	
	Area3	153.9875	0.1305	153.8750	154.0772	
Cr	Area1	81.0483	0.1514	80.9561	81.1404	100
	Area2	71.0258	0.1456	70.9372	71.1144	
	Area3	94.0241	0.1299	93.9450	94.1032	
Pb	Area1	28.0441	0.1374	27.9605	28.1277	100
	Area2	17.9791	0.1134	17.9101	18.0482	
	Area3	38.0250	0.1214	37.9474	38.1053	
Ni	Area1	43.0716	0.1281	42.9886	43.1815	100
	Area2	28.9966	0.1463	28.9076	29.0856	
	Area3	56.0325	0.1448	55.9388	56.1285	

Note: N—number of collected and analyzed samples from each area; SD—standard deviation; CI—confidence interval; MAL—Maximum allowable level; A1 area, the harbor area near Port of Tomis, the beach area (A2) near Costinesti Beach and the industrial area (A3) near Navodari.

**Table 9:** Estimated daily intake rates and hazard quotient of metals through mussel consumption in A1 (harbor), A2 (beach) and A3 (industrial).

Heavy Metal	EDI *			HQ			RfD [44,45] (mg/kg bw)
	A1	A2	A3	A1	A2	A3	
Cd	3 × 10^−5^	1.3 × 10^−5^	3.5 × 10^−4^	3 × 10^−2^	1.3 × 10^−2^	0.35	0.001
Cu	5.6 × 10^−4^	3.1 × 10^−4^	6.5 × 10^−4^	1.1 × 10^−3^	6.2 × 10^−4^	1.3 × 10^−3^	0.5
Zn	7.2 × 10^−3^	5 × 10^−4^	8.4 × 10^−4^	7 × 10^−4^	5 × 10^−4^	8.4 × 10^−4^	1
Cr	3.3 × 10^−3^	2.8 × 10^−4^	4.2 × 10^−4^	2.3 × 10^−2^	2 × 10^−3^	3 × 10^−3^	0.14
Pb	7 × 10^−5^	4.2 × 10^−5^	1 × 10^−4^	0.2	1.2 × 10^−2^	3 × 10^−2^	0.0035
Ni	1.3 × 10^−4^	7.2 × 10^−5^	3.1 × 10^−4^	6.5 × 10^−3^	3.6 × 10^−3^	1.5 × 10^−2^	0.02

EDI = Estimated daily intake (* for an adult with 60 kg body weight); HQ = Hazard quotient; RfD = Reference Dose.

## Data Availability

Not applicable.
